# The mediating role of coping styles in the relationship between perceived social support and antenatal depression among pregnant women: a cross-sectional study

**DOI:** 10.1186/s12884-022-04377-9

**Published:** 2022-03-08

**Authors:** Zhonglan Chen, Youping Li, Juan Chen, Xiujing Guo

**Affiliations:** 1 Evidence-Based Nursing Centre, West China Hospital, Sichuan University, Chengdu, 610041 Sichuan China; 2 West China School of Nursing, Sichuan University, Chengdu, 610041 Sichuan China; 3 Chinese Evidence-Based Medicine Centre, West China Hospital, Sichuan University, Chengdu, 610041 Sichuan China; 4 Mental Health Centre, West China Hospital, Sichuan University, Chengdu, 610041 Sichuan China; 5 Department of Obstetrics and Gynaecology, West China Second University Hospital, Sichuan University/Key Laboratory of Birth Defects and Related Diseases of Women and Childen, Ministry of Education, No. 37 Guo Xue Xiang, Chengdu, 610041 Sichuan China

**Keywords:** Antenatal depression, Positive coping styles, Negative coping styles, Social support

## Abstract

**Background:**

Antenatal depression (AD) is common in pregnant women and is associated with adverse outcomes for the mother, fetus, infant and child. The influencing factors of AD among pregnant women have been studied; however, the mechanisms of these factors remain unclear. This study was designed to examine the direct and serial mediating roles of coping styles in the relationship between perceived social support and AD among pregnant women.

**Methods:**

A cross-sectional study was conducted among 1486 pregnant women who registered to give birth at a tertiary hospital. A self-developed questionnaire was administered to obtain sociodemographic and obstetric data. The Perceived Social Support Scale (PSSS), Simplified Coping Style Questionnaire (SCSQ), and Edinburgh Postnatal Depression Scale (EPDS) were administered to measure the perceived social support, coping styles, and depressive symptoms of pregnant women, respectively. Multiple linear stepwise regression analysis was used, and then, the specific relationships among influencing factors were determined through structural equation modelling (SEM).

**Results:**

The prevalence of AD was 24.02%. The average scores of intrafamily support, extrafamily support, positive coping styles, negative coping styles and EPDS reported by pregnant women were 24.16 ± 3.09, 44.52 ± 6.16, 27.34 ± 4.89, 9.79 ± 3.82, and 7.44 ± 3.56, respectively. Multiple regression analysis showed that pregnant women with a higher level of intrafamily support exhibited a positive coping style and a decreased risk of AD. Compared with extrafamily support, the direct effect (-0.16 vs. -0.10, *P* < 0.05) and indirect effect of intrafamily support through coping styles (-0.028 vs. -0.027, *P* < 0.05) on AD were stronger. Two indirect pathways explained 17.46% of the variance in the EPDS scores.

**Conclusion:**

Higher social support decreased the likelihood of AD, not only directly but also through the mediating roles of coping styles. Social support should be strengthened, and positive coping styles should be advocated in every stage of pregnancy. Specifically, intrafamily support should be given more attention for pregnant Chinese women.

**Supplementary Information:**

The online version contains supplementary material available at 10.1186/s12884-022-04377-9.

## Background

Pregnancy is a challenging period for women, during which pregnant women experience a series of physical and mental changes [1]. These changes place substantial pressure on pregnant women and can lead to adverse outcomes such as AD, fatigue and cardiovascular disease [2, 3]. The prevalence of AD in Chinese women ranges from 18.5% to 28.5% [4–6]. Moreover, the reported prevalence of AD may be underestimated [7]. AD can lead to multiple and significant adverse effects and is associated with adverse birth and child outcomes such as premature birth, low birth weight, children's behavioural problems and adverse maternal outcomes such as suicide and postpartum depression [8–11].

China’s one-child policy ended on January 1, 2016, and three children have been advocated since May 31, 2021 [12]. Births to older mothers increased from 10.1% to 19.9% between 2011 and 2016 with the change of birth policy in China [13]. However, older maternal age is a risk factor associated with AD [14].

Perceived social support, defined as the perceived support of individuals through social ties with other individuals, groups and the larger community [15], was confirmed to be a protective factor for depression, which might indirectly lead to social adjustments, reduce stress, and thereby enhance physical and mental fitness [16–18]. However, whilst the importance of the level of social support received by pregnant women is recognized, the mechanisms by which this support exerts its effects remain unclear.

In addition to social support, poor coping was also the main predictor of depression regardless of race and population [19–22]. Coping styles refer to the cognitive and behavioural changes that result from the management of an individual’s specific external/internal stressors [23, 24]. Coping styles varied in the literature. They can be divided into positive and negative coping, problem-focused and emotion-focused coping, or approach coping and avoidance coping [25, 26]. Chinese and other international studies found that the coping style the pregnant women adopted directly affected their mental and physical health during pregnancy and after delivery [19, 27, 28]. Individuals who apply a negative coping style often exhibit distortion of thinking, and make negative appraisals and inappropriate self-evaluations (e.g., feeling their inability to tackle problems) [26]. Furthermore, accumulative evidence suggests that both social support and coping styles are associated with prenatal and postpartum depression [29, 30]. Pregnant women with lower perceived social support were more inclined to adopt avoidant coping styles, which in turn led to more severe depression [29], whilst pregnant women with good social support and positive coping styles were less prone to AD [30].

**Fig. 1:**
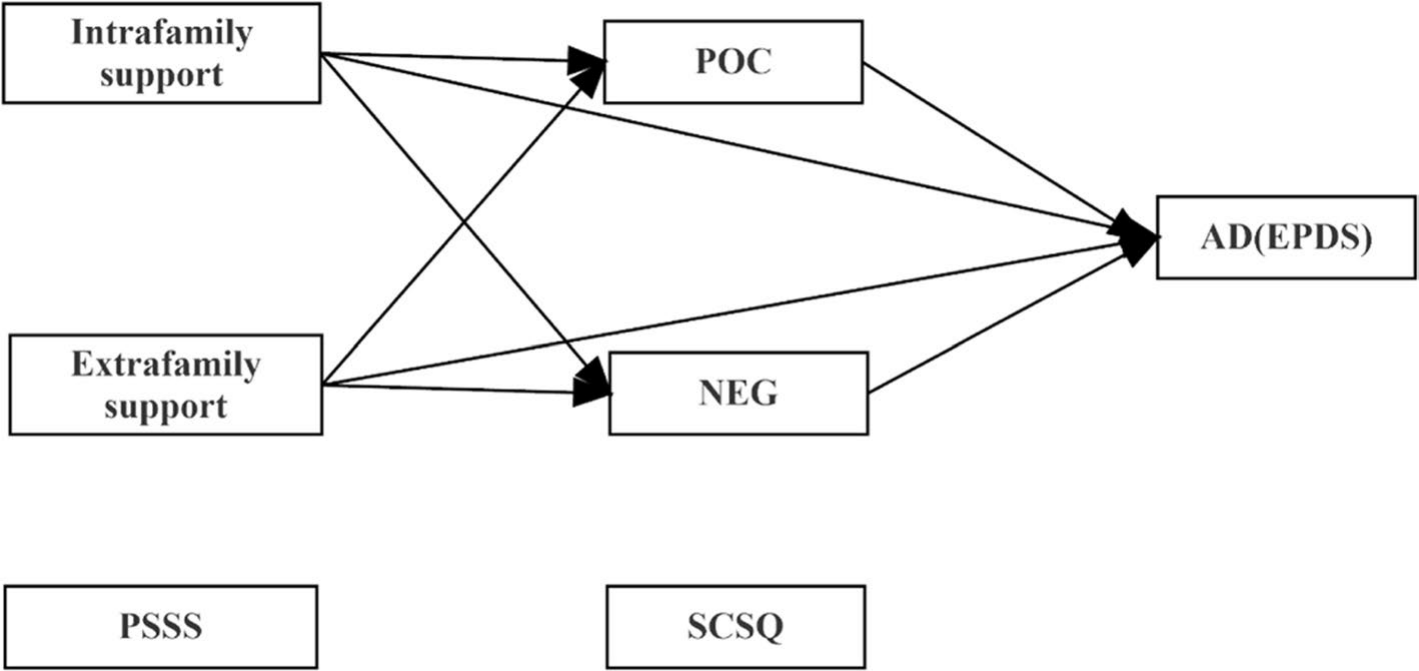
Hypothesized mediation effect model. Abbreviations: AD: Antenatal depression; EPDS: Edinburgh Postnatal Depression Scale; POC: Positive coping styles; NEG: Negative coping styles. PSSS: The Perceived Social Support Scale; SCSQ: Simplified Coping Style Questionnaire

Taken together, AD, coping styles and perceived social support are all interrelated. Thus, it is reasonable to hypothesize that coping styles may mediate the relationship between social support and AD. On the basis of our previous study that provided evidence that stronger family support may help improve the mental health of pregnant women [31], this study focused on the question of how intrafamily and extrafamily support affected antenatal depression through the mediation of coping styles. We used data collected from 1486 pregnant women to test the following three hypotheses: 1) perceived social support affected coping styles and the likelihood of AD; 2) coping styles increased/decreased the likelihood of AD; and 3) coping styles mediated the relationship between perceived social support and AD in pregnant women (Fig. 1).

The findings from this study will be of value for improving perinatal mental health services and designing interventions to reduce antenatal depression, thus leading to improvements in the mental and physical well-being of pregnant women.

## Methods

### Design and participants

This cross-sectional study was conducted at the outpatient department of the largest women's and children's hospitals in Western China, which is located in Cheng Du, Sichuan Province. Many women from Sichuan and other provinces of Western China come to this hospital to give birth, and approximately 15,000 babies are delivered in this hospital per year, which accounts for approximately 10% of the total annual deliveries in Cheng Du. Convenience sampling was adopted to enroll pregnant women who registered to give birth at this hospital.

The inclusion criteria were as follows: ① voluntary participation in research; ②age 18 years or older; ③ first appointment of that pregnancy in this hospital; and ④ ability to complete the study independently. The exclusion criteria were as follows: ① personal and family psychiatric history; and ②severe systemic diseases.

The ethics committee of West China Second University Hospital of Sichuan University approved the study protocol (No. 2019 (002)). The procedures used in this study adhered to the principles of the Declaration of Helsinki. All pregnant women signed informed consent forms before participation.

### Measurement

#### Basic characteristics

We used a self-developed questionnaire (Additional file 1) to collect basic characteristics from the pregnant women. The information included age, ethnicity (Han or ethnic minority), religion (yes or no), marital status, occupation (employed or not), place of residence (city or countryside), family income per month, and educational level. Obstetric data included gravity (primigravida or multigravida), gestational week, and pregnancy plan (planned natural conception, unintended natural conception, natural conception following fate, and assisted reproductive technology).

#### Perceived social support

Perceived social support was assessed with the Chinese version of the Perceived Social Support Scale (PSSS) [32, 33], which was developed by Zimet [32] and revised by Jiang QJ [33]. It has 12 items that assess how individuals perceive social support from their families (4 items), friends (4 items), and significant others (4 items), and these items can be analysed from internal and external aspects of the family (intrafamily support and extrafamily support). Each item is scored on a 7-point Likert scale (1 = very strongly disagree to 7 = very strongly agree), with a higher score representing stronger perceived social support. The good psychometric characteristics of this scale have been confirmed in Chinese populations [15, 34], the Cronbach’s alpha coefficient of the three domains was 0.88, and the test–retest reliability was 0.85 [35].

#### Coping styles

Coping styles were assessed with the Simplified Coping Style Questionnaire (SCSQ) [36], which was revised by Xie [36] based on the Ways of Coping Questionnaire designed by Folkman and Lazarus [37]. The questionnaire consists of 20 items that are divided into two subscales: positive coping styles (items 1–12) and negative coping styles (items 13–20). Behaviours such as actively talking and listening, seeing events from a favourable perspective, and seeking solutions to problems were considered positive coping styles. However, avoiding actions such as evading reality, depending on others to solve problems, and performing bodily harm activities were considered negative coping styles [30, 36]. Each item is graded on a 4-point scale ranging from 0 = never to 3 = always. A higher score on each subscale indicates a more frequent use of that coping style. The test–retest reliability and Cronbach’s alpha coefficient of the scale were 0.89 and 0.90, respectively [36, 38]. Previous studies reported good reliability of the Chinese version of the SCSQ among Chinese pregnant women [6, 30].

#### AD

The Edinburgh Postnatal Depression Scale (EPDS) was used to assess the participants’ self-reported depressive symptoms, which was developed by Cox [39]. The Chinese mainland version of this scale was validated by Wang et al. [40, 41]. It can be used to screen for postpartum depression as well as AD [40, 42]. The EPDS contains a total of 10 items, and each item is scored on a 4-point scale ranging from 0 to 3, with the total score ranging from 0 to 26. Higher scores indicate a higher possibility of depression [43]. The Cronbach’s alpha was 0.78, and the test–retest reliability was 0.90 [44]. A cutoff of 10 has been shown to be valid on the Chinese version of this scale [44].

### Data collection

Based on the variables in our study, considering the number of babies delivered in the hospital annually (approximately 15,000, approximately 1200 per month), we planned to enroll approximately 1500 eligible pregnant women in three months. Thereafter, we collected data from March 2019 to May 2019. To enroll more eligible pregnant women, we collected data at the obstetric care archive room of the outpatient department, where all pregnant women made an appointment of prenatal examination and delivery of that pregnancy in the hospital.

Before completing the questionnaire, all participants were instructed by uniformly trained staff. Approximately 15 to 20 min were required to complete all questionnaires.

### Statistical analysis

SPSS 25.0 and Amos 26.0 software (SPSS Inc., Chicago, IL, USA) were used for statistical analysis. Descriptive statistics were used to summarize the basic characteristics of the participants and the average PSSS, SCSQ and EPDS scores. Pearson correlation analysis was performed to determine the correlations among perceived social support, coping styles and depression. Scores on the EPDS and the two coping style dimensions were used as dependent variables and background variables in all three analyses, PSSS and SCSQ in the AD analysis, and PSSS in the positive/negative coping analyses were used as independent variables to perform multiple regression analysis. The inclusion level of the equation was 0.05, and the elimination level was 0.10. Structural equation modelling (SEM) was performed to analyse the path relationships between the measured variables. The goodness-of-fit index (GFI), comparative fit index (CFI), normed fit index (NFI), and incremental fix index (IFI) were used to evaluate the optimum of the model. Standardized path coefficients were used to analyse the effects of perceived social support and coping styles on AD. *P* < 0.05 was statistically significant (two-tailed test).

## Results

### General characteristics of participants and their scores of perceived social support, coping styles and AD

A total of 1551 pregnant women were invited to participate in the study, of which 1486 women participated and 65 women refused; the response rate was 95.81%. According to the cutoff value of 10, the prevalence of AD was 24.02%, and the average EPDS score was 7.44 ± 3.56 (total range: 0 to 26). The majority of the women had a college education level above (87.89%) and were employed (86.20%). Most of these women (90.44%) lived in cities, with a family income of more than 5000 Yuan per month. Among them, the majority of the pregnant women were primiparae (64.87%). The age of the pregnant women ranged from 20 to 44, with a total of 24.6% of the participants at advanced maternal age (≥ 35 years at the time of childbirth [45]). A slightly lower proportion was planned natural conceptions (48.38%). The mean gestational week was 14 (SD = 3.54), ranging from 9 to 36 weeks.

As shown in Table 1, the pregnant women who were employed had a higher score on intrafamily support (t = 3.10, *p* < 0.01), extrafamily support (t = 3.59, *p *< 0.01), and positive coping styles (t = 3.31, *p *< 0.01) and a lower score on depression (t = -3.03, *p* = 0.006). Moreover, those who lived in the city had lower scores on the EPDS and higher scores on the other four measurements (intrafamily support, extrafamily support, positive and negative coping styles), *p* < 0.01. Furthermore, higher scores on the EPDS and lower scores on the other four measurements were also observed in participants with lower educational levels (*p* < 0.05). There were no significant differences in the EPDS, PSSS and SCSQ scores between different marital statuses.

**Table 1:** Perceived social support, coping styles and EDPS scores among participants with different characteristics (*n* = 1486)

**Characteristics**	**N**	**%**	**Perceived social support **[33]		**Coping styles** [15]		**Antenatal depression**
			**Intrafamily Support (Mean ± SD)**	**Extrafamily Support (Mean ± SD)**	**Positive Coping (Mean ± SD)**	**Negative Coping (Mean ± SD)**	**EDPS **[38]** (Mean ± SD)**
Nationality							
Ethnic minority	42	2.83	24.71 ± 3.18	45.50 ± 5.17	28.95 ± 4.11	10.12 ± 4.51	6.62 ± 2.45
Han	1444	97.17	24.14 ± 3.09	44.49 ± 6.19	27.29 ± 4.90	9.78 ± 3.80	7.47 ± 3.59
			*p *= 0.24	*p* = 0.29	*p* = 0.03	*p* = 0.58	*p* = 0.13
Religion							
No	1406	5.38	24.18 ± 3.07	44.60 ± 6.16	27.30 ± 4.91	9.73 ± 3.82	7.42 ± 3.57
Yes	80	95.36	23.74 ± 3.36	43.10 ± 6.01	27.93 ± 4.53	10.83 ± 3.71	7.89 ± 3.45
			*p* = 0.21	*p* = 0.035	*p* = 0.27	*p* = 0.013	*p* = 0.25
Marital status							
First marriage	1418	95.42	24.17 ± 3.10	44.54 ± 6.19	27.32 ± 4.89	9.78 ± 3.84	7.45 ± 3.53
Remarriage	64	4.31	24.10 ± 2.98	44.11 ± 5.88	27.21 ± 4.94	9.98 ± 3.68	7.39 ± 4.27
Spinsterhood	4	0.27	23.00 ± 1.41	41.75 ± 3.77	31.00 ± 2.71	11.75 ± 1.89	7.50 ± 3.42
			*p* = 0.34	*p* = 0.74	*p* = 0.49	*p *= 0.67	*p* = 0.67
Educational level							
Secondary school and below	180	12.11	23.12 ± 3.49	41.71 ± 6.46	25.78 ± 5.65	8.97 ± 4.15	8.12 ± 3.79
University and above	1306	87.89	24.30 ± 3.00	44.90 ± 6.02	27.56 ± 4.74	9.91 ± 3.77	7.35 ± 3.52
			*p* = 0.003	*p* < 0.001	*p* < 0.001	*p* = 0.012	*p* = 0.011
Employment status							
Housewife	205	13.80	23.54 ± 3.54	43.09 ± 6.29	26.29 ± 5.34	9.81 ± 3.76	8.14 ± 4.04
Employed	1281	86.20	24.26 ± 3.00	44.75 ± 6.12	27.50 ± 4.80	9.79 ± 3.84	7.33 ± 3.47
			*p* = 0.001	*p* = 0.002	*p *= 0.001	*p *= 0.93	*p* = 0.002
Place of residence							
Town or countryside	142	9.56	23.42 ± 3.52	42.23 ± 6.80	25.53 ± 5.24	9.03 ± 4.08	8.30 ± 4.02
City	1344	90.44	24.24 ± 3.03	44.76 ± 6.05	27.52 ± 4.82	9.87 ± 3.79	7.35 ± 3.50
			*p* = 0.008	*p* < 0.001	*p* < 0.001	*p* = 0.007	*p* = 0.002
Family income per month (RMB)							
< 5000	242	16.29	23.56 ± 3.32	42.72 ± 6.73	26.30 ± 4.85	9.48 ± 3.65	7.95 ± 3.66
5001 ~ 8000	491	33.04	23.98 ± 3.18	44.51 ± 6.00	26.95 ± 5.19	9.73 ± 3.76	7.20 ± 3.51
8001 ~ 10,000	337	22.68	24.28 ± 3.01	44.79 ± 6.12	27.61 ± 4.68	10.02 ± 3.89	7.47 ± 3.61
> 10,000	416	27.99	24.61 ± 2.79	45.34 ± 5.83	28.18 ± 4.56	9.87 ± 3.94	7.41 ± 3.51
			*p* < 0.001	*p* < 0.001	*p* < 0.001	*p* = 0.299	*p* = 0.006
Gravity							
Primigravida	964	64.87	24.17 ± 3.07	44.78 ± 5.96	27.16 ± 4.86	9.63 ± 3.67	7.59 ± 3.53
Multigravida	522	35.13	24.14 ± 3.13	44.03 ± 6.50	27.65 ± 4.93	10.10 ± 4.08	7.18 ± 3.61
			*p* = 0.84	*p *= 0.03	*p* = 0.07	*p* = 0.02	*p* = 0.04
Pregnancy plan							
Planned NC	719	48.38	24.43 ± 2.94	45.02 ± 6.10	27.65 ± 5.55	9.76 ± 3.69	7.31 ± 3.29
Untended NC	280	18.84	23.66 ± 3.34	43.91 ± 6.10	27.01 ± 5.25	9.80 ± 3.66	7.86 ± 3.91
NC following fate	312	21.06	24.05 ± 3.06	44.39 ± 6.27	27.07 ± 5.09	9.99 ± 4.21	7.34 ± 3.62
ART	174	11.71	24.01 ± 3.23	43.73 ± 6.18	26.99 ± 5.30	9.68 ± 3.93	7.60 ± 3.94
			*p* = 0.01	*p* = 0.01	*p *= 0.18	*p* = 0.405	*p* = 0.09
Total	1486		24.16 ± 3.09	44.52 ± 6.16	27.34 ± 4.89	9.79 ± 3.82	7.44 ± 3.56

Abbreviations: *ART* Assisted reproductive technology, *EPDS* Edinburgh Postnatal Depression Scale, *NC* Natural conception, *PSSS* The Perceived Social Support Scale, *SD* Standard deviation

### Correlation analysis between social support, coping styles and AD

As shown in Table 2, both intrafamily support and extrafamily support were positively and significantly associated with positive coping styles (r ranged from 0.32 to 0.36, *P* < 0.01). These variables were also negatively and significantly associated with the EPDS (r ranged from -0.27 to -0.24, *P* < 0.01). Positive coping styles were negatively and significantly associated with the EPDS (r = -0.22, *P* < 0.01). However, negative coping styles were positively and significantly associated with the EPDS (r = 0.15, *P *< 0.01).

**Table 2:** Pearson correlation analysis between PSSS, coping styles and AD (*n* = 1486)

**Variables**		**Extrafamily support**	**Intrafamily support**	**Negative coping styles**	**Positive coping styles**	**EPDS scores**
Extrafamily support	r	1.000				
Intrafamily support	r	0.647**	1.000			
Negative coping styles	r	0.110**	0.076**	1.000		
Positive coping styles	r	0.360**	0.317**	0.264**	1.000	
EPDS scores	r	-0.244**	-0.265**	0.153**	-0.222**	1.000

^**^ *p* < 0.01. Abbreviations: *AD* Antenatal depression, *EPDS* Edinburgh Postnatal Depression Scale, *PSSS* The Perceived Social Support Scale

### Effects of demographic factors, perceived social support and coping styles on AD

As Table 3 showed, negative coping styles were the strongest predictor for AD (B = 0.23, *p* < 0.01), followed by positive coping styles (B = -0.19, *p* < 0.01) and intrafamily support (B = -0.15, *p* < 0.01). These variables explained 15.0% of the variance in the AD score among the pregnant women (R^2^ = 0.15) and could significantly affect the AD score (*P *< 0.001).

**Table 3:** Effect of sociodemographic factors, PSSS and coping styles on AD (*n* = 1486)

	Unstandardized coefficient		Standardization factor	t	P	Collinearity statistics	
	B	SE	B			Tolerance	VIF
Constant	17.00	1.05		16.16	0.00		
Age	-0.05	0.02	-0.05	-2.21	0.03	0.97	1.03
Place of residence	0.34	0.19	0.04	1.76	0.08	0.97	1.03
Intrafamily support	-0.18	0.04	-0.15	-4.80	0.00	0.57	1.75
Extrafamily support	-0.06	0.02	-0.10	-3.09	0.00	0.55	1.83
Positive coping styles	-0.14	0.02	-0.19	-6.98	0.00	0.80	1.26
Negative coping styles	0.22	0.02	0.23	9.30	0.00	0.93	1.08
R^2^ = 0.15, F = 43.06, *P* < 0.001							

Abbreviations: *AD* Antenatal depression, *PSSS* The Perceived Social Support Scale, *VIF* Variance inflation factor

### Effects of demographic factors and perceived social support on coping styles

As shown in Tables 4 and 5, the observed variables could explain 16.0% of the variance in positive coping styles (R^2^ = 0.16) and 3.4% of the variance in negative coping styles (R^2^ = 0.03). Extrafamily support was the strongest predictor for positive coping styles (B = 0.26, *p* < 0.01), followed by intrafamily support (B = 0.13, *p* < 0.01). Interestingly, extrafamily support was the strongest predictor for negative coping styles (B = 0.11, *p* < 0.01). In summary, both intrafamily and extrafamily support affected positive coping style scores; however, extrafamily support scores affected both positive coping and negative coping scores (*p* < 0.01).

**Table 4:** Effect of sociodemographic factors, PSSS on positive coping styles (*n* = 1486)

	Unstandardized coefficient		Standardization factor	t	P	Collinearity statistics	
	B	SE	B			Tolerance	VIF
Constant	8.43	1.48		5.71	0.00		
Age	0.11	0.03	0.09	3.62	0.00	0.96	1.04
Religion	0.63	0.36	0.04	1.77	0.08	0.98	1.02
Place of residence	-0.64	0.27	-0.06	-2.39	0.02	0.92	1.09
Family income per month (RMB)	0.28	0.11	0.06	2.60	0.01	0.92	1.09
Intrafamily support	0.21	0.05	0.13	4.19	0.00	0.58	1.73
Extrafamily support	0.21	0.03	0.26	8.38	0.00	0.57	1.75
R^2^ = 0.16, F = 47.9, *P* < 0.001							

Abbreviations: *PSSS* The Perceived Social Support Scale, *RMB* Renminbi, *VIF* Variance inflation factor

**Table 5:** Effect of sociodemographic factors, PSSS on negative coping styles (*n* = 1486)

	Unstandardized coefficient		Standardization factor	t	P	Collinearity statistics	
	B	SE	B			Tolerance	VIF
Constant	0.94	1.41		0.67	0.50	0.99	1.01
Age	0.07	0.02	0.07	2.83	0.00	0.87	1.15
Education level	0.95	0.31	0.08	3.07	0.00	0.98	1.02
Religion	0.66	0.30	0.06	2.19	0.03	0.88	1.13
Employed	0.07	0.03	0.06	2.05	0.04	0.96	1.04
Extrafamily support	0.07	0.02	0.11	4.13	0.00	0.99	1.01
R^2^ = 0.03, F = 8.48, *P* < 0.001							

Abbreviations: *PSSS* The Perceived Social Support Scale, *VIF* Variance inflation factor

### The mediating effects of coping styles between social support and AD

**Fig. 2:**
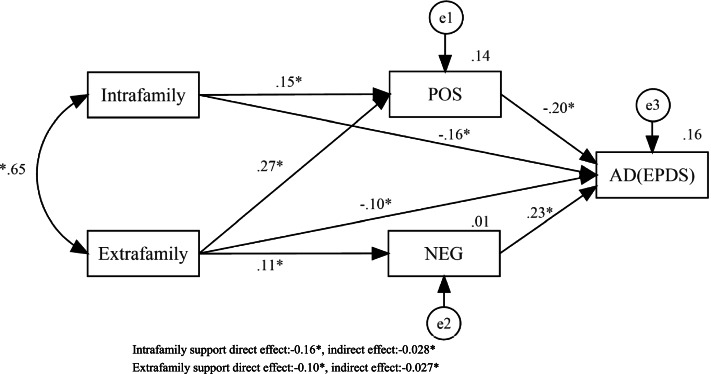
Mediating effects among perceived social support (independent variable), coping styles (mediator), and EPDS scores (outcome variable) (*n* = 1486). *p < 0.01. Abbreviations: AD: Antenatal depression; EPDS: Edinburgh Postnatal Depression Scale; POC: Positive coping styles; NEG: Negative coping styles; e represents the residual error

As depicted in Fig. 2, intrafamily and extrafamily support significantly and directly predicted positive coping styles (standardized direct effect ranged from 0.15 to 0.27, *P* < 0.01). Intrafamily support, extrafamily support and positive coping styles directly and significantly predicted AD (standardized direct effect ranged from -0.20 to -0.10, *P* < 0.01). Negative coping scores directly and significantly predicted AD (standardized direct effect = 0.23, *P* < 0.01). Interestingly, extrafamily support significantly and directly predicted both positive coping styles (standardized direct effect = 0.27, *P* < 0.01) and negative coping styles (standardized direct effect = 0.11, *P* < 0.01). Moreover, intrafamily support reduced AD via the mediating effect of positive coping styles [standardized indirect effect was -0.028, 95% CI: (-0.015, -0.045)]. However, extrafamily support reduced AD via the counterbalance of positive and negative coping styles [standardized indirect effect was -0.027, 95% CI: (-0.007, -0.049)]. In summary, two indirect pathways explained 17.46% of the variance in the EPDS scores. The model indicated a reasonable fit (χ2/df = 3.92, CFI = 0.935, IFI = 0.936, GFI = 0.977, NFI = 0.934).

## Discussion

To the best of our knowledge, this is the first study on the effects of perceived social support and coping styles on AD in pregnant Chinese women since the birth policy changed in 2016. The incidence of AD was 24.02% in our study. As depicted in Table 3, among the variables in the study, negative coping styles were the strongest predictor, followed by positive coping styles. As depicted in Fig. 2, the direct effect (-0.16 vs. -0.10) and indirect effect (-0.028 vs. -0.027) of intrafamily support on AD were stronger than those of extrafamily support.

In our study, only 24.02% of respondents reported depressive symptoms, and the average EPDS score was generally low, which was different from other studies on Chinese and Ethiopians [46, 47]. This may have occurred because the participants of this study had better family situations, education levels and social status. A systematic review of studies that were conducted in predominantly high-income countries estimated that the point prevalence of major depressive disorder was between 3.1% and 4.9% during pregnancy [48]. However, another study found that the pooled prevalence of depression among African pregnant women [49] was 26.3%, with some countries having prevalence up to 39.5%. Additionally, depression was shown to be significantly associated with economic difficulties, poor support from relatives, and poor obstetric history. These results imply that there are differences in the prevalence of AD among pregnant women from different cultures and different demographics.

This study found that intrafamily support and extrafamily support are both direct significant negative predictors for AD, indicating that pregnant women who perceived higher support from within and outside the family were less likely to have a diagnosis of AD. Our results partially supported Hypothesis 1 that higher perceived social support decreased the likelihood of AD and are consistent with previous studies [30, 31, 50]. Furthermore, accumulative evidence indicates that while the incidence of AD increases during earthquakes and the COVID-19 pandemic, social support remains an effective protective factor [51–54]. Social support therefore can be an important intervention target for pregnant women under any circumstance.

Moreover, our study found that the direct and indirect effects of intrafamily support were stronger than those of extrafamily support. Similar results were found in Chinese and other East Asian pregnant women. Research by Shen and Yeatts indicated that family support had a significant predictive effect on women's life satisfaction [55]. Research by Qiu and Shi et al. [56, 57] found that poor family support and a poor relationship with the husband and mother-in law were significantly associated with AD. Accumulative evidence suggests [50] that family support might be much more important in East Asian populations than in their Western counterparts. Consequently, support from family members may be much more important than extrafamily support for pregnant women in the Chinese culture. Health care professionals should encourage family members to participate in prenatal health care with pregnant women for their well-being.

This study also found that positive coping style was a direct significant negative predictor for AD, indicating that pregnant women who used positive coping strategies were less likely to have a diagnosis of AD. Negative coping styles were a direct significant positive predictor for AD, indicating that the pregnant women who used negative coping strategies were more likely to have a diagnosis of AD. Our results are consistent with previous studies [30, 58] and supported Hypothesis 2, that coping styles affected the likelihood of AD in this study, indicating that a holistic approach for AD screening and related identifiable factors such as coping styles also should be advocated for pregnant women at a high risk of AD. Women with negative styles might avoid thinking about or confronting a stressful situation through rumination, self-blame, disengagement or social isolation [59, 60]. However, positive coping is associated with better psychological adjustment during pregnancy [61]. One possibility is the cognitive reappraisal mechanisms of women in interpreting stress [62]. Accordingly, cognitive reappraisal may be functional in changing coping styles to accommodate stressful circumstances, leading to more effective coping with the uncontrollable aspects of a stressful event [63]. Therefore, clinical health care workers should pay more attention to pregnant women who have negative coping styles and help them develop positive coping styles.

The present study also explored the mediation effects of coping styles between perceived social support and AD after controlling for covariates. In other words, this study found that in addition to the direct effects, perceived social support also affected the likelihood of AD indirectly through coping styles. As Fig. 2 showed, the path relationships of “intrafamily support-positive coping styles- AD during pregnancy” and “positive/negative coping styles-AD during pregnancy” were established. Negative coping styles had the greatest impact on AD, followed by positive coping styles, intrafamily support and extrafamily support during pregnancy. According to Borcherding KE [64, 65], coping styles might mediate the relationship between stress and depression. The current research supported Hypothesis 3, confirmed this point and clearly explained the path relationship. A higher level of perceived social support decreased the likelihood of AD not only directly but also indirectly by enhancing positive coping styles. Pregnant women who perceive more social support are more likely to develop high self-esteem and subjective well-being, have higher self-efficacy to adapt to stress [27], and adopt appropriate coping styles with the care of family and friends. Thus, a high level of social support minimizes the adversity caused by stress, such as depression and other physical and mental illnesses [25].

Based on the findings, we suggest that AD prevention programmes should be strengthened in the perinatal mental health service for pregnant Chinese women and can be focused on strengthening pregnant women’s social support system and training them how to get help and cope with pregnancy positively. Specifically, intrafamily support should be given more attention. The participation of family members such as husbands and mothers-in law in perinatal health care should be encouraged.

## Conclusion

In addition to the direct effects, perceived social support affected antenatal depression indirectly through coping styles. Improving intrafamily and extrafamily support is all regarded as efficient, as these types of support also help pregnant women strengthen their active coping skills. Specifically, intrafamily support should be given more attention among the Chinese population. Among these variables in the study, negative coping styles were the strongest predictor, indicating the crucial role of adopting a holistic approach in screening AD for pregnant women. For pregnant women with negative coping styles, interventions should be focused on helping them adopt actions associated with positive coping rather than negative coping styles. These findings can help to provide constructive suggestions for intervention targets for AD prevention and perinatal mental health services.

### Limitations and strengths

We conducted a large-scale study, found useful results for pregnant women, and identified the path relationship between perceived social support and AD. Nevertheless, there were still some limitations when interpreting our findings. First, the research was a cross-sectional research design, which reflected the relationships among variables only in a specific time period. Second, the participants of the study included only pregnant women in one hospital, and most of the pregnant women who registered in the hospital mainly represented those of Han nationality living in cities. Therefore, these results cannot be generalized to women in other parts of China. In the future, additional investigations should be carried out to validate the model in other demographic categories, such as minorities and those in rural areas, as contextual and sociocultural factors may exert effects on AD. Furthermore, as both social support and antenatal depression are risk factors postpartum, the effect of social support and its mechanisms still need to be studied.

## Supplementary Information

**Additional file 1. **General sociodemographic and obstetric information questionnaire

## Data Availability

All data supporting our findings are presented in the manuscript; the datasets used and/or analysed during the current study are available from the corresponding author upon reasonable request.

## Abbreviations

AD: Antenatal depression
ART: Assisted reproductive technology
CI: Confidence interval
EPDS: Edinburgh Postnatal Depression Scale
PSSS: Perceived Social Support Scale
SCSQ: Simplified Coping Style Questionnaire
SD: Standard deviation
SEM: Structural equation modelling
