# Supplementary material for: The mediating role of coping styles in the relationship between perceived social support and antenatal depression among pregnant women: a cross-sectional study
Source: BMC Pregnancy Childbirth. 2022 Mar 8;22:188. doi: 10.1186/s12884-022-04377-9 (PMC8902494; doi:10.1186/s12884-022-04377-9)
Supplement: Supplementary file 1 — Additional file 1. General sociodemographic and obstetric information questionnaire [file 12884_2022_4377_MOESM1_ESM.doc]

**General sociodemographic and obstetric information questionnaire**

This part is about your sociodemographic and obstetric situation. Please answer the following questions below by selecting a choice or filling in the blanks.

Q1 What is your date of birth (year/month)__________？

Q2 What is your ethnicity?

■Han

■ethnic minority, and my ethnicity was_____

Q3 What is your religion?

■None

■Yes, and my religion was_____

Q4.What is your education level？

■High school or lower

■University and above

Q5.What is your marital status？

■First marriage

■Remarriage

■Spinsterhood

Q6. What is your employment status?

■Housewife

■Employed

Q7. Where you lived?

■Town or countryside

■City

Q8. How mch was your family income per month?

■< 5000

■5001~8000

■8001~10000

■> 10000

Q9 What is your gestational age?__________weeks

Q10. Is this pregnancy planned?

■Planned natural conception

■Untended natural conception

■Natural conception following fate

■Assisted reproductive technology

Q11. What is your current pregnancy?

■Primigravida

■Multigravida
